# Rotavirus-Associated Myositis in a 12-Year-Old Child

**DOI:** 10.7759/cureus.84946

**Published:** 2025-05-28

**Authors:** Arisa Hayashi, Mika Ukai, Shoichiro Kanda, Keiichi Takizawa, Akiko Kinumaki

**Affiliations:** 1 Pediatrics, The University of Tokyo, Tokyo, JPN

**Keywords:** acute myositis, complication of infection, creatine kinase (ck), pediatric infection, rotavirus

## Abstract

Rotavirus infection is a common cause of pediatric gastroenteritis, often self-limited but occasionally associated with extraintestinal complications. This report describes a 12-year-old vaccinated girl who presented with acute gastroenteritis and an incidental elevation of serum creatine kinase (CK). Despite the absence of muscle pain or weakness during hospitalization, laboratory findings indicated skeletal muscle involvement, with elevated CK and aldolase levels, and a predominance of CK-MM isoenzyme. Myocardial involvement and rhabdomyolysis were ruled out. The patient recovered with supportive care, and CK levels normalized after discharge, although she reported mild post-discharge fatigue. Rotavirus-associated myositis is rare and likely underrecognized, particularly in young children who may not verbalize muscle symptoms. This case highlights the importance of considering myositis in patients with elevated CK during rotavirus infection, even in the absence of overt muscular symptoms.

## Introduction

Rotavirus infection is one of the leading causes of acute gastroenteritis, particularly in infants and young children [1]. It can cause severe diarrhea and vomiting, potentially leading to dehydration. Although rotavirus infection was previously associated with high global morbidity and mortality, the introduction of rotavirus vaccines has markedly reduced disease incidence. The effectiveness of vaccination has been widely demonstrated, and many countries have reported a substantial decline in the number of cases in parallel with increased vaccination coverage [2].

While rotavirus infection is primarily a gastrointestinal viral illness, extraintestinal complications may occasionally occur. Reported complications include myocarditis [3], encephalitis [4], respiratory involvement [5], and pancreatitis [6]. Some cases require intensive care management or follow a fatal course, necessitating vigilant clinical monitoring. Herein, we report a pediatric case of rotavirus infection complicated by myositis, along with a review of the relevant literature.

## Case presentation

The patient was a 12-year-and-7-month-old girl with no significant past medical history, perinatal complications, or developmental abnormalities. She had received all routine immunizations, including the rotavirus vaccine. Specifically, she was administered two doses of the monovalent live attenuated oral rotavirus vaccine (Rotarix®, GlaxoSmithKline) at two and three months of age, in accordance with the recommended immunization schedule in Japan. These doses were administered on time, and no catch-up immunization was required. She had not taken any oral medications prior to the onset of symptoms. Four to two days prior to presentation, her mother and younger sister, with whom she lived, developed symptoms consistent with infectious gastroenteritis. Two days before presentation, the patient began experiencing soft stools, followed by frequent watery diarrhea (approximately once per hour), nausea, abdominal pain localized initially around the umbilicus and later shifting to the right lower quadrant, and fever with a maximum recorded temperature of 39°C.

**Figure 1:**
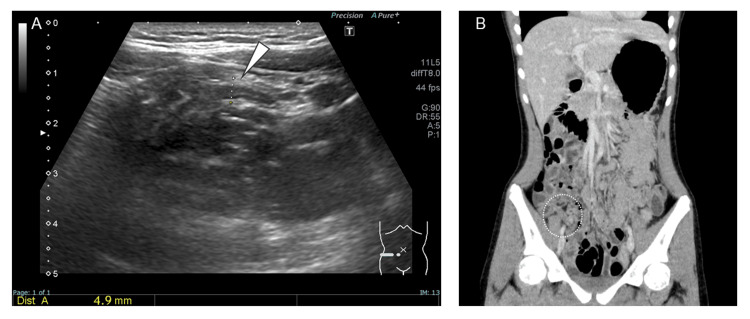
Appendiceal imaging findings A: Abdominal ultrasonography showing a longitudinal view of the appendix, indicated by the white arrowhead. The maximum outer diameter is 4.9 mm, which is within the normal range (<6 mm). No wall thickening is observed. B: Contrast-enhanced abdominal computed tomography image. The appendix is located within the white dotted circle. There is no evidence of appendiceal enlargement, wall thickening, or periappendiceal inflammation.

Upon examination, her vital signs were as follows: temperature of 38.9°C, heart rate of 134 bpm, blood pressure of 128/72 mmHg, and oxygen saturation of 98% on room air. She was alert and fully oriented. Spontaneous pain was noted in the right lower quadrant of the abdomen, but no rebound tenderness was present. Acute appendicitis was suspected; however, abdominal ultrasonography and contrast-enhanced computed tomography revealed no evidence of appendiceal enlargement (Figure 1).

Laboratory investigations (Table 1) showed a white blood cell count of 8,800/μL and a mildly elevated C-reactive protein level of 1.14 mg/dL. In the clinical context of persistent high fever, abdominal pain, and vomiting, laboratory evaluation included measurement of serum creatine kinase (CK) to screen for potential muscle or myocardial involvement. CK was markedly elevated at 7,435 U/L, which prompted further evaluation, including serum myoglobin measurement. Aspartate aminotransferase was 82 U/L, and lactate dehydrogenase was 289 U/L. Serum creatinine and acid-base status were within normal limits, and urinalysis revealed no abnormalities. A stool antigen test was performed on day 2 of illness and was positive for rotavirus. The test was conducted using the RapidTesta® Rota-Adeno II, an immunochromatographic assay kit for simultaneous detection of rotavirus and adenovirus antigens (Sekisui Medical Co., Ltd., Tokyo, Japan). According to the manufacturer, the kit has a sensitivity of 100%, a specificity of 99.3%, and an overall agreement rate of 99.5%. Due to the persistence of abdominal symptoms, she was admitted for further management.

**Table 1:** Laboratory findings on admission CLEIA: chemiluminescent enzyme immunoassay, MDA5: melanoma differentiation-associated protein 5, TIF1: transcription intermediary factor 1

Parameters	Value	Reference range
Complete blood count		
White blood cell count (per μL)	8,800	3,800-10,100
Hemoglobin (g/dL)	12.5	11.9-14.9
Platelet count (per μL)	303,000	180,000-440,000
Serum chemistry		
Total protein (g/dL)	7.3	6.3-7.8
Albumin (g/dL)	4.2	3.8-4.7
Total cholesterol (mg/dL)	136	125-230
Sodium (mmol/liter)	138	138-144
Potassium (mmol/liter)	3.4	3.6-4.7
Chloride (mmol/liter)	102	102-109
Urea nitrogen (mg/dL)	13.9	6.8-19.2
Creatinine (mg/dL)	0.60	0.39-0.52
C-reactive protein (mg/dL)	1.14	<0.3
Lactate dehydrogenase (U/liter)	289	145-270
Alanine aminotransferase (U/liter)	34	9-28
Aspartate aminotransferase (U/liter)	82	15-30
Creatine kinase (U/L)	7435	45-210
Creatine kinase-MB (U/L)	14	0-12
Myoglobin (ng/mL)	484	0-65
High-sensitivity cardiac troponin I (pg/mL)	<10.0	0-26.2
Aldolase (U/L)	62.8	2.7-7.5
Thyroid function tests		
Thyroid-stimulating hormone (μIU/mL)	0.22	0.61-4.23
Free thyroxine (ng/dL)	1.41	0.71-1.69
Free triiodothyronine (pg/mL)	2.1	1.72-3.44
Myositis-specific autoantibodies		
Anti-Jo-1 antibody (CLEIA)	<1.0	0-9.9
Anti-aminoacyl-tRNA synthetase antibody	<5.0	0-24.9
Anti-MDA5 antibody	<4	0-31
Anti-Mi-2 antibody	<5	0-53
Anti-TIF1-γ antibody	<5	0-31
Urinalysis		
Specific gravity	1.019	1.006-1.030
pH	6.5	5.0-7.5
Ketones	Negative	Negative
Blood	+/-	Negative
Protein	Negative	Negative
WBC	Negative	Negative
Urinary myoglobin (ng/mL)	<2.0	0-2.0

**Figure 2:**
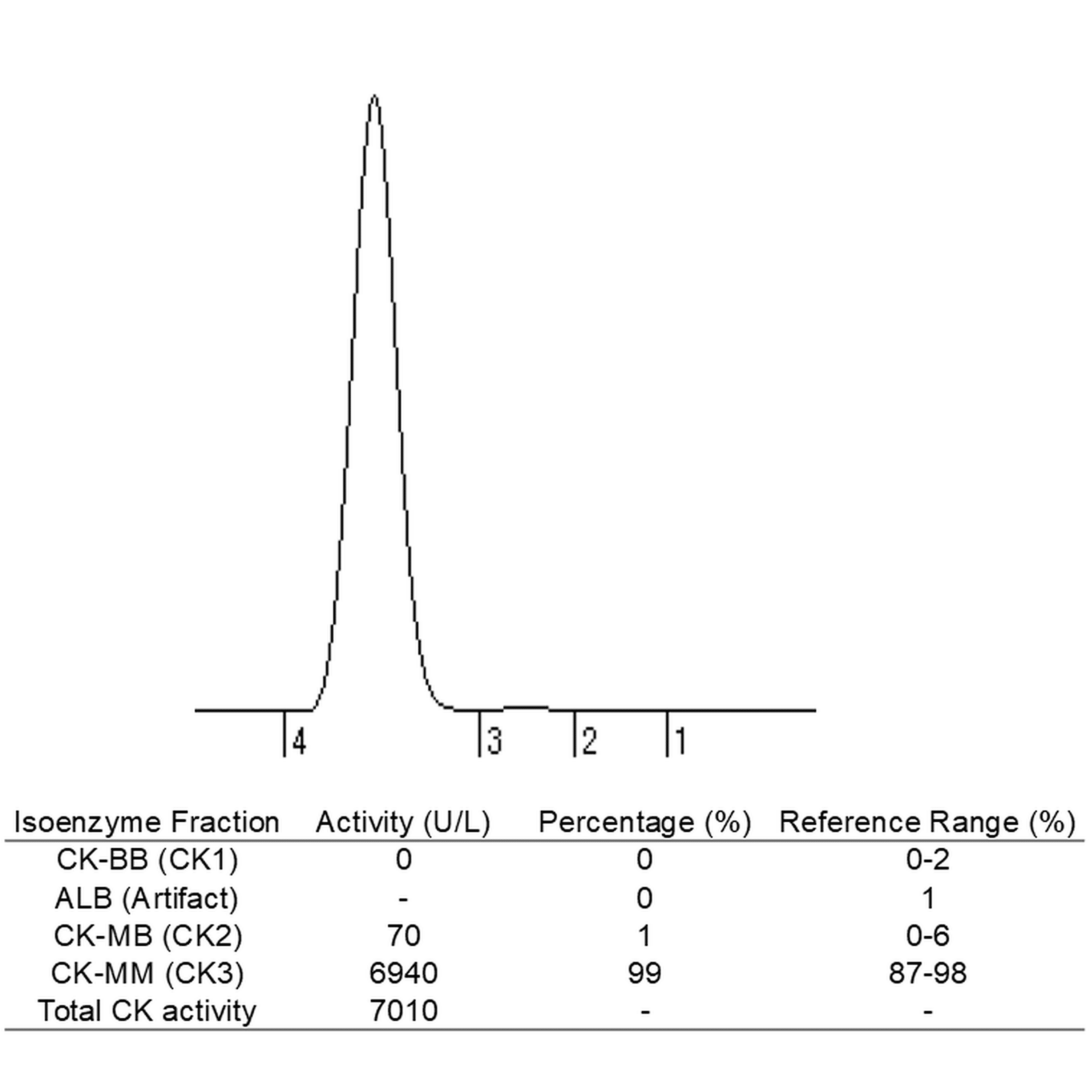
CK isoenzyme profile The CK isoenzyme analysis revealed a predominant CK-MM fraction. No macro-CK was detected. CK: creatine kinase

Further evaluation of the elevated CK revealed a CK-MB level of 14 U/L and a negative high-sensitivity troponin I test. Electrocardiography was unremarkable. CK isoenzyme analysis (Figure 2) demonstrated a predominance of CK-MM (99%), with no detectable macro-CK, indicating a skeletal muscle origin. Aldolase was elevated at 62.8 U/L. Urinary myoglobin was negative, ruling out rhabdomyolysis. Thyroid function was normal, and all autoantibodies associated with autoimmune myositis were negative. Complement levels were within normal limits. Based on these findings, a diagnosis of rotavirus-associated myositis was made.

**Figure 3:**
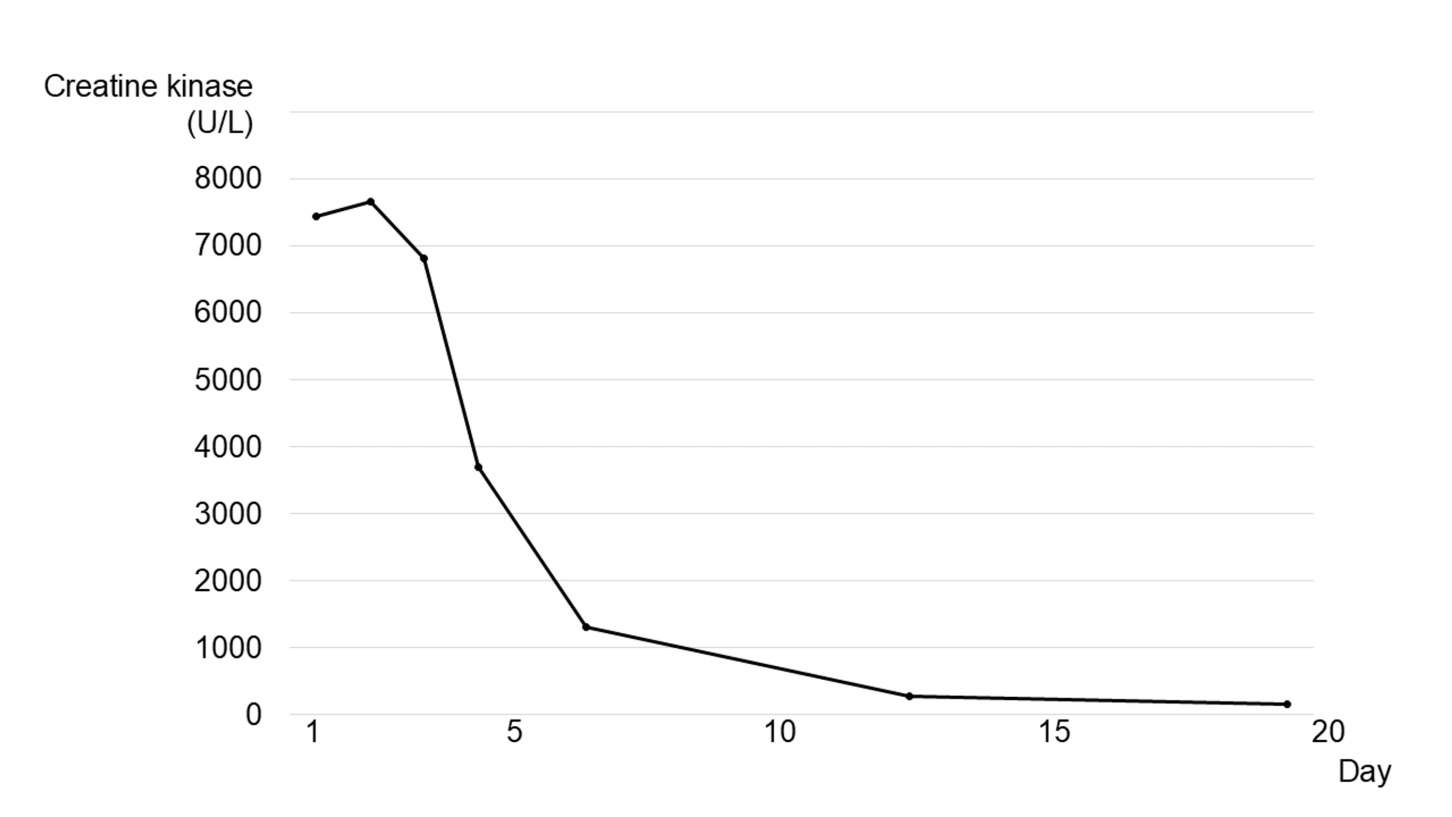
Temporal trend of serum CK levels Serum CK levels showed a transient increase following admission but declined rapidly with conservative management. Normalization was confirmed during outpatient follow-up. CK: creatine kinase

Supportive care with bowel rest and intravenous fluids was initiated. By the following day, the patient's abdominal symptoms began to improve. Although CK levels peaked transiently at 7,667 U/L, they subsequently declined spontaneously. With the resolution of gastrointestinal symptoms, she was discharged on day 6 of hospitalization. Follow-up outpatient testing confirmed normalization of CK levels (Figure 3).

Throughout the clinical course, the patient did not report any muscle pain. Muscle weakness had not been apparent during bed rest in the hospital. However, following discharge and return to daily activities, she noted transient difficulty exerting muscle strength compared to her baseline for several days.

## Discussion

This report describes a pediatric case of rotavirus infection in which an incidental elevation in serum CK was observed following the onset of gastroenteritis symptoms. After excluding other differential diagnoses, the patient was diagnosed with rotavirus-associated myositis. Muscle weakness was mild, myalgia was absent, and CK levels normalized spontaneously without the need for specific medical intervention.

Rotavirus is a leading cause of acute gastroenteritis in children; however, extraintestinal manifestations such as hepatic dysfunction [7] and encephalitis [4] have also been reported. Among these, myositis, myocarditis, and rhabdomyolysis are of particular clinical concern, as they can present with elevated serum CK levels and, in severe cases, may require intensive care management [4]. In the present case, there were no abnormalities suggestive of myocardial involvement, no findings indicative of rhabdomyolysis, and macro-CK was not detected, supporting the diagnosis of myositis.

Myositis associated with rotavirus infection is considered rare, with only sporadic case reports available. Reported cases predominantly involve children over the age of two and are characterized by the development of muscle symptoms and CK elevation a few days after the onset of gastrointestinal symptoms [8]. The age of our patient was consistent with previously reported cases. Notably, in infants and younger children, muscular symptoms may go unreported due to limited verbal expression, suggesting that the actual incidence of this complication may be underrecognized.

The pathogenesis of rotavirus-associated myositis remains unclear, but two major mechanisms have been proposed: direct viral invasion of muscle tissue and indirect immune-mediated injury. Regarding the former, the detection of rotavirus RNA in cerebrospinal fluid and blood has been documented, suggesting the possibility of viremia and dissemination to extraintestinal organs [9]. In terms of the latter, cytokine-mediated inflammation, including elevated levels of interleukin-6 and tumor necrosis factor-alpha [4], as well as transient hypocomplementemia [10,11], has been implicated in muscle damage. The delayed rise in CK levels observed in some cases may support the immune-mediated hypothesis. In our case, CK elevation followed gastrointestinal symptoms; however, complement levels remained within the normal range throughout the course.

Both host and viral factors may contribute to the development of myositis in the context of rotavirus infection, although the specific determinants remain unidentified. The role of particular viral subtypes in the pathogenesis of myositis is also unclear [8]. In this report, viral genotyping was not performed, which represents a limitation and an area for future investigation. Regarding host factors, there was no family history of neuromuscular disease, and CK levels normalized without intervention, suggesting no identifiable predisposing condition in this patient.

## Conclusions

Rotavirus-associated myositis remains an uncommon and underrecognized entity, particularly in vaccinated children with otherwise typical presentations of gastroenteritis. This case underscores the importance of considering muscular involvement when unexplained elevations in CK are observed, even in the absence of overt myalgia or muscle weakness. In clinical practice, selective testing for rotavirus may be warranted in children presenting with acute gastroenteritis and unexpected hyperCKemia, especially during outbreaks or when multiple family members are affected. Targeted surveillance and accumulation of similar cases, along with virological and immunological analyses, will be essential to further clarify the pathophysiological mechanisms and guide appropriate management.
